# Resting state neurophysiology of agonist–antagonist myoneural interface in persons with transtibial amputation

**DOI:** 10.1038/s41598-024-63134-4

**Published:** 2024-06-12

**Authors:** Laura A. Chicos, D. Rangaprakash, Shriya S. Srinivasan, Samantha Gutierrez-Arango, Hyungeun Song, Robert L. Barry, Hugh M. Herr

**Affiliations:** 1 Biomechatronics Group, Massachusetts Institute of Technology, Media Lab, Cambridge, MA 02139 USA; 2 K. Lisa Yang Center for Bionics, Massachusetts Institute of Technology, Cambridge, MA 02139 USA; 3 Athinoula A. Martinos Center for Biomedical Imaging, Department of Radiology, Massachusetts General Hospital, Charlestown, MA 02129 USA; 4 Department of Radiology, Harvard Medical School, Boston, MA 02115 USA; 5 Harvard–MA Institute of Technology Division of Health Sciences and Technology, Cambridge, MA 02139 USA; 6 John A. Paulson School of Engineering and Applied Sciences, Harvard University, Allston, MA 02134 USA; 7 McGovern Institute for Brain Research, Massachusetts Institute of Technology, Cambridge, MA 02139 USA

**Keywords:** Neural circuits, Tissue engineering

## Abstract

The agonist–antagonist myoneural interface (AMI) is an amputation surgery that preserves sensorimotor signaling mechanisms of the central-peripheral nervous systems. Our first neuroimaging study investigating AMI subjects conducted by Srinivasan et al. (2020) focused on task-based neural signatures, and showed evidence of proprioceptive feedback to the central nervous system. The study of resting state neural activity helps non-invasively characterize the neural patterns that prime task response. In this study on resting state functional magnetic resonance imaging in AMI subjects, we compared functional connectivity in patients with transtibial AMI (*n* = 12) and traditional (*n* = 7) amputations (TA). To test our hypothesis that we would find significant neurophysiological differences between AMI and TA subjects, we performed a whole-brain exploratory analysis to identify a seed region; namely, we conducted ANOVA, followed by *t*-test statistics to locate a seed in the salience network. Then, we implemented a seed-based connectivity analysis to gather cluster-level inferences contrasting our subject groups. We show evidence supporting our hypothesis that the AMI surgery induces functional network reorganization resulting in a neural configuration that significantly differs from the neural configuration after TA surgery. AMI subjects show significantly less coupling with regions functionally dedicated to selecting where to focus attention when it comes to salient stimuli. Our findings provide researchers and clinicians with a critical mechanistic understanding of the effect of AMI amputation on brain networks at rest, which has promising implications for improved neurorehabilitation and prosthetic control.

## Introduction

Every year in the United States, approximately 150,000 patients undergo lower-extremity amputations due to various factors, including diabetes mellitus, peripheral vascular disease, neuropathy, and trauma^1^*.* The financial cost of lower-extremity amputation in the U.S. due to dysvascular etiology alone, which accounts for the majority (82%) of amputations, exceeds $4.3 billion annually^2,3^. Over half of all amputations occur at the transtibial level, or below the knee^4^. The traditional surgical technique employed for nearly all of these amputations involves transecting nerves and burying them within the residuum. This traditional amputation (TA) often results in patients experiencing painful neuromas, phantom limb pain, and pathological brain activity^5,6^. One critical drawback with the TA is that agonist–antagonist muscle pairs are cleaved, which limits muscle spindle and Golgi tendon organ-based proprioceptive afferent signals from reaching the central nervous system (CNS). Subsequently, patients rely heavily on visual feedback to guide their prosthesis during free-space movements instead of natural afferent feedback. While there is still no clear mechanistic understanding as to how sensorimotor phantom limb representation modulates pain processing, the leading hypothesis for decades has been that phantom limb pain may be the result of maladaptive plasticity in response to the loss of afferent input^7,8^.

**Figure 1:**
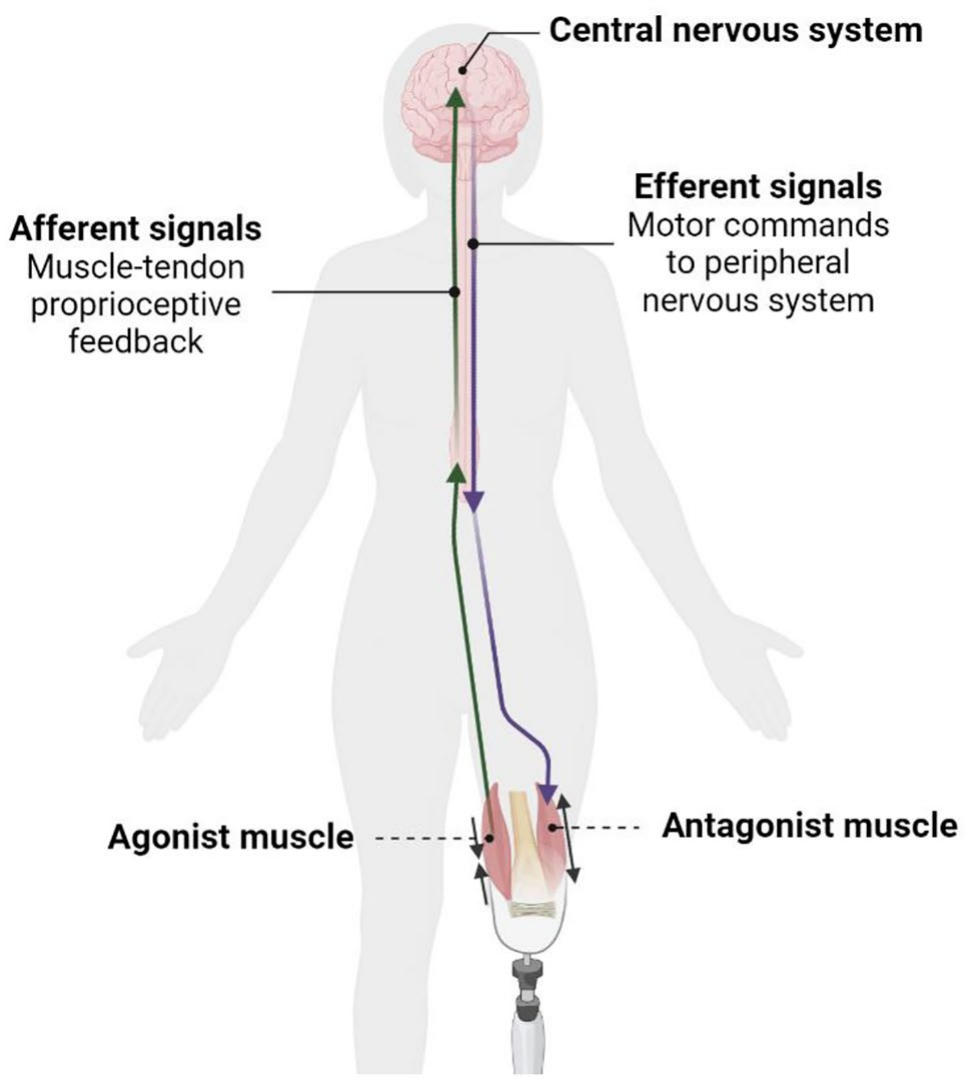
The Agonist–Antagonist Myoneural Interface (AMI) amputation architecture. An agonist–antagonist muscle pair is surgically created in the residual limb for coupled fascicle dynamics. The purple arrow represents the pathway of efferent signals responsible for transmitting motor commands instantiated in the central nervous system to the peripheral nervous system to initiate and guide movement. The green arrow represents the pathway of afferent signals responsible for conveying muscle–tendon proprioceptive feedback from the residual limb to the central nervous system.

The agonist–antagonist myoneural interface (AMI) amputation promotes physiological function of the central-peripheral sensorimotor signaling mechanisms by reestablishing agonist–antagonist muscle pairs (Fig. 1)^9^. The effect of the restoration of afferent feedback due to the AMI amputation on CNS plasticity in relation to prosthesis embodiment, as well as phantom limb sensation and pain, are potentially impactful future research areas^10^.

The first neuroimaging study investigating AMI subjects focused on task-based neural signatures, and showed evidence of AMI promoting proprioceptive feedback to the CNS in terms of functional activation in proprioceptive centers compared to persons with TA^11^. Subjects with an AMI amputation had higher position differentiation task scores than TA subjects on tasks requiring motor control and proprioception, presumably due to the reinstituted afferent feedback. Furthermore, the strongly coupled relationship between the sensorimotor network and the visual cortex was associated with subjects that reported weaker non-painful phantom sensations*.* Building on these important discoveries, it is also important to gain a mechanistic understanding of how AMI affects intrinsic brain networks and plasticity at rest, to further substantiate the efficacy of the AMI surgical architecture and its overall impact on the brain, prosthesis control, embodiment, and quality of life.

Resting state functional magnetic resonance imaging (rsfMRI), or imaging during a task-free state, is an important tool for understanding neurophysiological states without confounding variability from task compliance or performance within and between subjects. RsfMRI helps in non-invasively probing intrinsic, spontaneous functional networks by quantifying co-fluctuations in the blood oxygenation level dependent (BOLD) signal between two spatially-distinct regions of interest (ROI), which is called functional connectivity (FC)^12^. These correlated fluctuations are most prominent at low frequencies (< 0.1 Hz) and prime task response^13^. There have been a few rsfMRI studies examining brain network-level functional reorganization after TA; for instance, one study showed changes in FC between sensorimotor and subcortical structures over time since amputation^14,15^. However, there are no existing studies on resting state FC signatures in transtibial AMI subjects and their differences with TA subjects. We address this critical gap in this study. The work by Srinivasan et al*.* (2020) found that the AMI architecture promotes proprioceptive feedback during tasks, and potentially decreases visuomotor dependence compared to persons with a TA architecture. As such, in this exploratory resting state analysis, we built upon our lab’s previous work to discover brain mechanisms that are impacted during the resting state. We hypothesized that the AMI amputation induces neuroplastic reorganization of functional networks that significantly varies from the TA in terms of FC maps. To test this hypothesis, we employed a combination of standard as well as robust, cutting-edge fMRI processing methods to investigate seed-based connectivity (SBC) in AMI subjects compared to TA subjects.

## Results

The results of this study demonstrate notable group-level neurophysiological differences in FC between transtibial AMI and TA groups. Specifically, we focused on a seed within the salience network, which we identified as statistically significant for further investigation using SBC analysis. Our SBC analysis revealed that AMI subjects exhibited decreased connectivity with the salience network seed in particular cluster-level inferences when compared to TA subjects.

**Figure 2:**
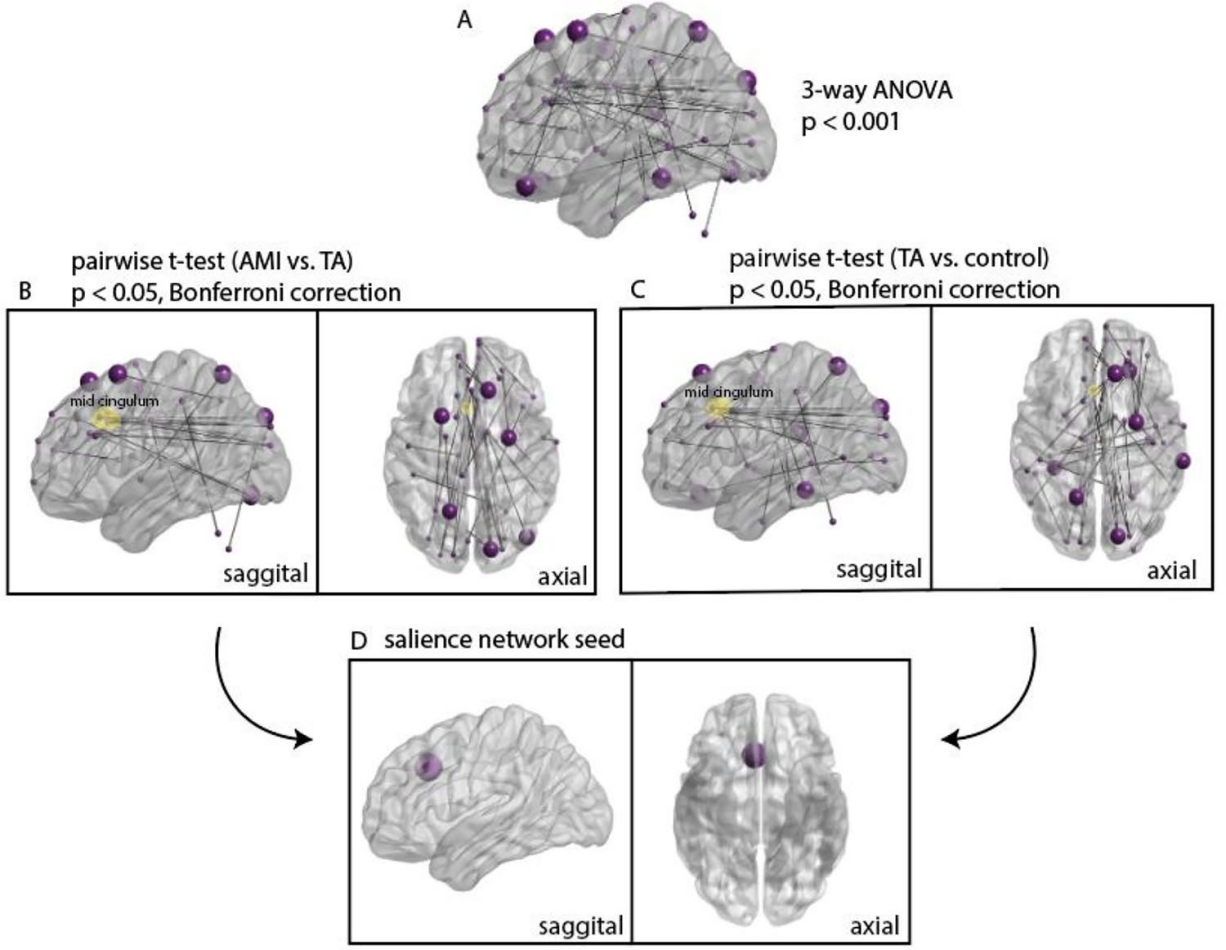
Whole-Brain Exploratory Analysis and ROIs selected for further investigation in a seed-based connectivity analysis. **(A)** Significant connections after a three-way ANOVA (p^unc^ < 0.001) between all three subject conditions. **(B)** Significant pairwise connections (*p* < 0.05, Bonferroni correction) between AMI and TA groups from the subset of significant connections after the three-way ANOVA. **(C)** Significant pairwise connections (*p* < 0.05, Bonferroni correction) between TA and control groups from the subset of significant connections after the three-way ANOVA. The node highlighted in the mid-cingulum represents the seed region in the salience network. **(D)** The salience network seed (spherical radius = 10 mm) chosen due to its uniquely high node degree and connectivity in both AMI vs. TA and TA vs. control pairwise connections.

Our goal was to investigate SBC in AMI, but, without a priori knowledge of potentially relevant seeds, we first assayed the rsfMRI data in an exploratory whole-brain analysis. To highlight the time series that were significantly correlated among any pair of the three subject conditions, a three-way ANOVA (p^unc^ < 0.001) was performed (Fig. 2A). Pairwise *t*-tests (*p* < 0.05, Bonferroni correction) were then performed between each subject condition to determine a seed region according to the following criteria: node degree and implication in more than one pairwise comparison. In both AMI vs. TA (Fig. 2B) and TA vs. biologically-intact control (Fig. 2C) comparisons, a region located on the mid-cingulum and part of the salience network (Fig. 2D) was associated with the largest number of significant connections (highest node degree). Therefore, we carried out further SBC analyses with this cingulate subregion associated with the salience network as the seed, which we henceforth refer to as the “salience network seed”. No region on the motor cortex was associated with a significant number of connections with this method, though we performed a speculative analysis with a motor cortex seed identified via meta-analysis (Fig. S1) in the supplementary materials (see result in Fig. S2).

### Neurophysiological Differences between *AMI* and TA Subjects

Seed-to-whole-brain FC was estimated separately with each of the identified seeds, and between-group differences were inferred from cluster-level inferences (Table 1) obtained by thresholding voxel-based connectivity spatial parametric maps (p^unc^ < 0.001 cluster-defining threshold and p^FDR^ < 0.001 cluster-level threshold). With the salience network seed, three significant cluster-level inferences were made (Table. 1).

**Table 1:** Table of the significant cluster-level differences (cluster-level threshold: p^FDR^ < 0.001, cluster-defining threshold: p^unc^ < 0.001) between AMI and TA subjects from the resting state SBC analysis. The table contains ROIs in relation to their FC with the salience network seed. Enumerated in the table is the region in which a given cluster is situated, based on the Harvard–Oxford atlas default in the CONN toolbox, each cluster’s centroid in MNI space, each cluster’s size, as well as the cluster-level and cluster-defining p-value thresholds. Abbreviations: FP = frontal pole, FO = frontal operculum, SMG = supramarginal gyrus, AG = angular gyrus, OP = occipital pole, LOC = lateral occipital cortex.

Salience network seed					
ROI spherical radius = 10 mm					
	Cluster region	Centroid (MNI Space)	Cluster Size	Cluster-defining p-unc	Cluster-level p-FDR
		AMI > TA			
	FP, FO	(-48, + 32, -14)	369	0.000001	< 0.000001
	SMG, AG	(+ 46, -38, + 28)	227	0.000259	0.000024
	OP, LOC	(-26, -86, + 32)	218	0.000694	0.000026

**Figure 3:**
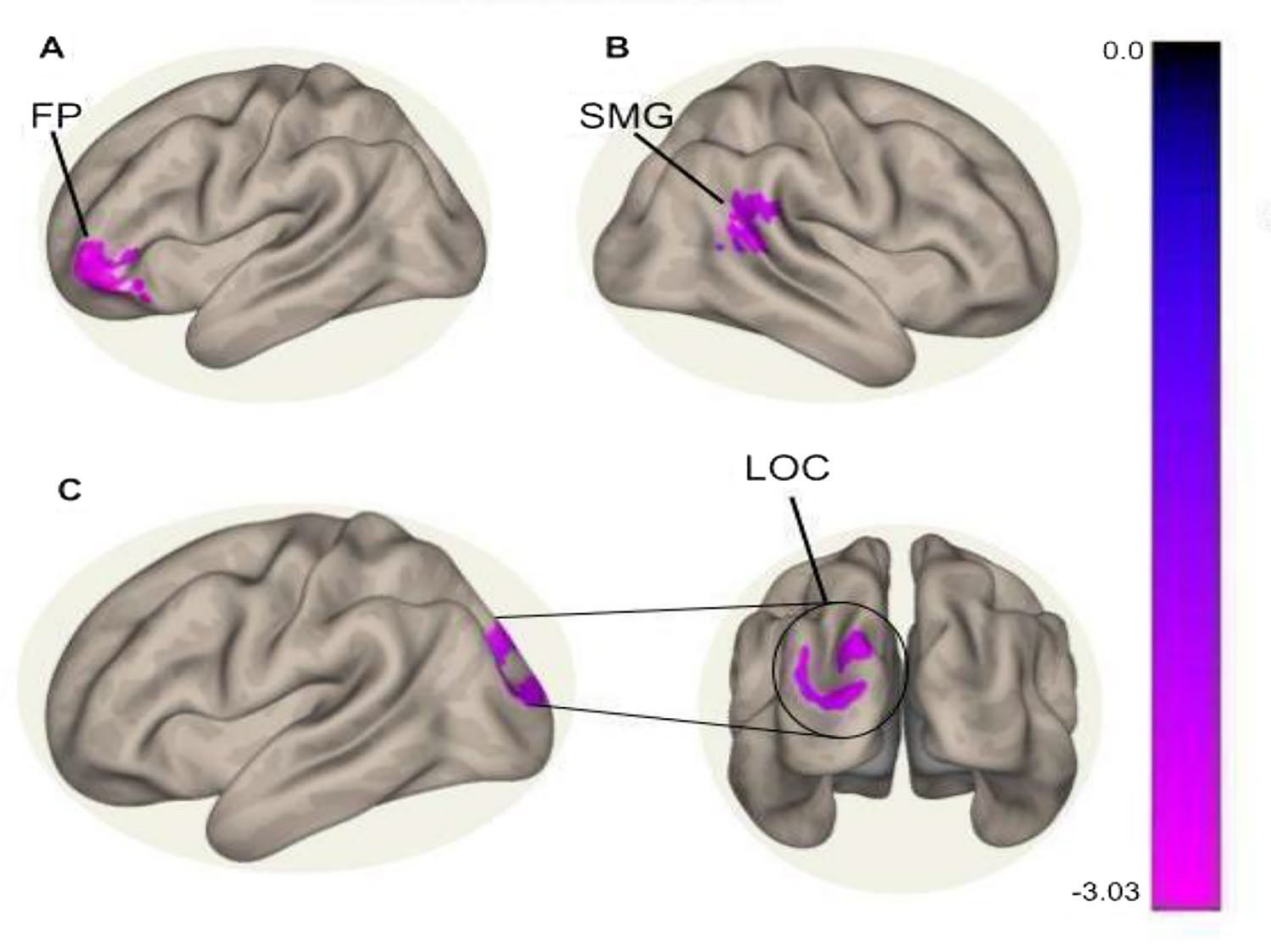
Results from the seed-based connectivity analysis with a between-subjects contrast of AMI > TA, controlling for age and head motion. The colorbar represents the Z-score detailing the extent of group differences in each cluster-level inference. Negative Z-scores indicate lower connectivity in the AMI cohort. The **(A)** frontal pole cluster, **(B)** supramarginal gyrus cluster, and **(C)** occipital lobe cluster had stronger connectivity with the salience network seed in the TA group.

The largest cluster (size = 369 voxels) in the frontal pole (Fig. 3A) showed decreased FC with the salience network in AMI subjects compared to TA subjects (p^unc^ < 0.001 cluster-defining threshold, p^FDR^ < 0.001 cluster-level threshold). The second largest cluster (size = 227 voxels) in the posterior supramarginal gyrus and the angular gyrus (Fig. 3B) similarly showed decreased FC with the salience network in AMI compared to TA subjects (p^unc^ < 0.001 cluster-defining threshold, p^FDR^ < 0.001 cluster-level threshold). Lastly, part of the occipital pole and lateral occipital cortex formed the third cluster (size = 218 voxels) (Fig. 3C), which exhibited significantly decreased FC with the salience network seed in AMI compared to TA subjects (p^unc^ < 0.001 cluster-defining threshold, p^FDR^ < 0.001 cluster-level threshold).

## Discussion

The goal of this study was to investigate group-level neurophysiological differences in FC between transtibial AMI and TA groups to obtain a mechanistic understanding of the brain network reorganization that occurs in the AMI paradigm. We hypothesized that the AMI surgery will induce reorganization of functional brain networks that significantly varies from those in the TA cohort. Indeed, we found several key neurophysiological differences between AMI and TA cohorts, particularly that AMI subjects showed significantly less coupling with regions functionally dedicated to selecting where to focus attention when it comes to salient stimuli. Findings from this study provide researchers and clinicians with a critical mechanistic understanding of the effect of AMI amputation on brain networks while not performing any extraneous task. Our findings provide neurobiological markers for assessing network reorganization after surgery, as well as identify important regions that have undergone rewiring post-operationally and their possible cognitive correlates that could be the focus of future neurorehabilitation efforts and research studies towards improved neuroprosthesis control.

Our results from the SBC analysis illustrated decreased connectivity between the salience network seed and frontal pole regions in a map contrasting AMI and TA cohorts. Menon et al^16^. have demonstrated that the salience network is involved in the filtering and detection of salient stimuli, or, in other words, deciding what to focus the brain’s limited perceptual and cognitive resources on based upon the subset of sensory data available^17^. The frontal pole, which lies in the prefrontal cortex, helps plan and organize movement, making decisions about which actions should be used for different situations^18^. The lower connectivity between these salience and frontal pole regions in AMI subjects may be due to the reinstituted proprioceptive feedback from intact agonist–antagonist muscle pairs or related to the speculation that AMI subjects rely less than TA subjects on their visual stream during motor control. Indeed, brain areas functionally associated with choosing which salient stimuli to focus on and motor execution are less intrinsically coupled, suggesting that AMI subjects dedicate fewer cognitive resources to planning movement than TA subjects. We also observed less coupling between clusters in the visual cortex and the salience network seed in AMI subjects, which would further corroborate the speculation that AMI subjects assign less salience to their visual stream than TA subjects^19–21^. Additionally, the cluster lying primarily on the supramarginal gyrus had decreased connectivity to the salience network seed in AMI subjects. The supramarginal gyrus plays a role in interpreting sensory data and in the perception of space and limb location, as well as identifying postures and gestures of other people as part of the mirror neuron system^22,23^. Thus, the reduced coupling between these spatially distinct regions in AMI subjects may be motivation to explore possible neural correlates of fMRI connectivity to neural resource allocation while a subject is exposed to proprioceptive and sensory data, or while their mirror neuron system is activated in a comparison with TA subjects.

Research looking at the cognitive load of amputees is important for prosthesis development and rehabilitation because the increased cognitive burden and neural fatigue associated with prosthesis use can lead to their abandonment^24,25^. The neural fatigue brought on by excessive cognitive load is impacted by factors such as reduced proprioception and phantom sensations, maladaptive plasticity, and lack of embodiment, which are all induced by the changes in neural schema caused by amputation^26–28^. In this research study, the reconfiguration of intrinsic brain networks, which prime task response, suggest that there may be a measurable reduction in cognitive burden during motor performance tasks in the AMI cohort compared to the TA cohort. To this effect, the current study provides motivation to more precisely study the impacts of the AMI surgery on cognitive burden and neural fatigue during prosthesis use and training.

A strength of our approach is the use of the NORDIC denoising technique, which significantly reduced the thermal noise in functional images and provided an approximate twofold increase in the statistical power. A limitation that needs to be recognized when assessing generalizability includes the sample size; however, at the time of data collection, the AMI amputation was only being performed by one surgeon in the world at Brigham and Women’s Hospital in Boston, MA, so the AMI cohort was as large as possible. Today, additional surgeons across the United States and the world are performing the AMI surgery, allowing future studies to rely on more surgically homogeneous cohorts. Finally, we did not left–right flip our patient data to align affected hemispheres because of cortical asymmetry (see Table S1 for amputation laterality). For instance, the left and right halves of the salience network are functionally not mirror images of each other and thus, flipping the brains would have blurred hemisphere-specific characteristics and corrupted the results^29^. As another example, spatial attention, which is relevant to proprioception and motor tasks, is predominantly processed in the right hemisphere in most people^30^. Subsequently, we did not have conclusive findings from analyzing the significant cluster-level differences between AMI or TA cohorts and controls (Table S2).

To summarize, our findings suggest that the rsfMRI connectivity patterns following the AMI surgery may lead to improved prosthetic control and increased sense of artificial limb embodiment. That is, AMI subjects show significantly less rsfMRI coupling with regions functionally dedicated to selecting where to focus attention when it comes to salient stimuli. These findings contribute a mechanistic understanding of FC differences between AMI and TA cohorts in the intrinsic brain networks that prime task response. These research contributions have implications for neurorehabilitation of persons with transtibial amputation and, more broadly, for clinical translation in the field of orthopedic surgery. For instance, this study could inform future research looking at the progression of plasticity for TA patients receiving an AMI revision surgery with regards to the effects on prosthetic device adaptation and utility. Future research could examine the ways in which the AMI’s neurophysiological effects correlate with prosthesis control and embodiment representation, as well as how clinicians may be able to predict rehabilitation outcomes and adapt to improve them with prosthesis training^31,32^. Future research on the neurophysiological impacts of the AMI surgical architecture should also extend these results across different combinations of agonist–antagonist muscle constructs in patients with both upper and lower limb amputation.

## Materials and methods

### Participants and study design

This research was performed in accordance with relevant guidelines and regulations. All participants were recruited in a non-blinded fashion and provided written informed consent prior to imaging. The protocol and procedures for functional neuroimaging were approved by the Mass General Brigham (formerly Partners HealthCare) Institutional Review Board (protocol no. 2017P002635).

The participants that underwent neuroimaging included unilateral lower limb AMI amputees (*n* = 12), TA (*n* = 7), and biologically-intact controls (*n* = 10). Within the AMI cohort, the majority of the scans were on patients with a transtibial AMI amputation, save for one patient with a transfemoral AMI. Similarly, within the TA cohort, the majority of the scans were on patients with transtibial standard amputations, save for one patient with a transfemoral amputation. As the cortical representations for the thigh and calf regions of the lower leg are maintained within a 2–3 mm radius from each other, which is approximately one functional voxel, we posited that the transfemoral outlier was sufficiently negligible and categorized the cohort according to the majority (i.e. as transtibial amputees)^33^. To the best of our ability, TAs were selected to be amputation-to-scan-time-matched and age-matched to AMI amputees and biologically-intact controls. The matching criteria prioritized amputation to scan time (± 13 month variance; SD, ± 6.15 months) and age (± 7 years variance; SD, 4.16 years), similar to that of prior studies that looked at neural plasticity following amputation^34–36^. Further details about subject demographics, dominant side, amputation to scan time, age and sex can be found in Table S1.

The AMI amputation paradigm of the subjects in this study consisted of an AMI construct for both the subtalar and ankle joints^37,38^. The subtalar AMI construct mechanically linked the tibialis posterior and the peroneus longus muscle. The ankle AMI construct mechanically linked the lateral gastrocnemius to the tibialis anterior muscle. In each construct, a tendon harvested from the amputated ankle joint at the time of amputation was passed through a synovial canal to link the muscle pair. TAs underwent standard amputation as per vascular and orthopedic protocols.

### Data acquisition

Structural and functional MRI data were collected in a 3 T scanner at the Athinoula A. Martinos Center for Biomedical Imaging at the Massachusetts General Hospital (MGH). The 3 T Connectome 1.0 scanner, based on the Siemens Skyra system, had the following customized parameters: Gmax = 300 mT/m “connectome” gradients and a slew rate of 200 mT/m/ms. Head motion was restricted by foam padding placed strategically inside the custom 64-channel array coil. Anatomical data was collected with a T1-weighted magnetization-prepared rapid acquisition gradient echo (MPRAGE) sequence with the following parameters: 1-mm isotropic resolution; 208 slices; flip angle, 7°; repetition time (TR), 2530 ms; echo time (TE), 1.61 ms; generalized autocalibrating partial parallel acquisition (GRAPPA) factor, 4; acquisition time, 3 min and 40 s. RsfMRI data were acquired using a 2D echo planar imaging (EPI) sequence with the following parameters: 2-mm isotropic voxels; 68 slices; flip angle, 41°; TR, 1080 ms; TE, 30 ms; 8-min run (445 volumes).

### Preprocessing and denoising

**Figure 4:**
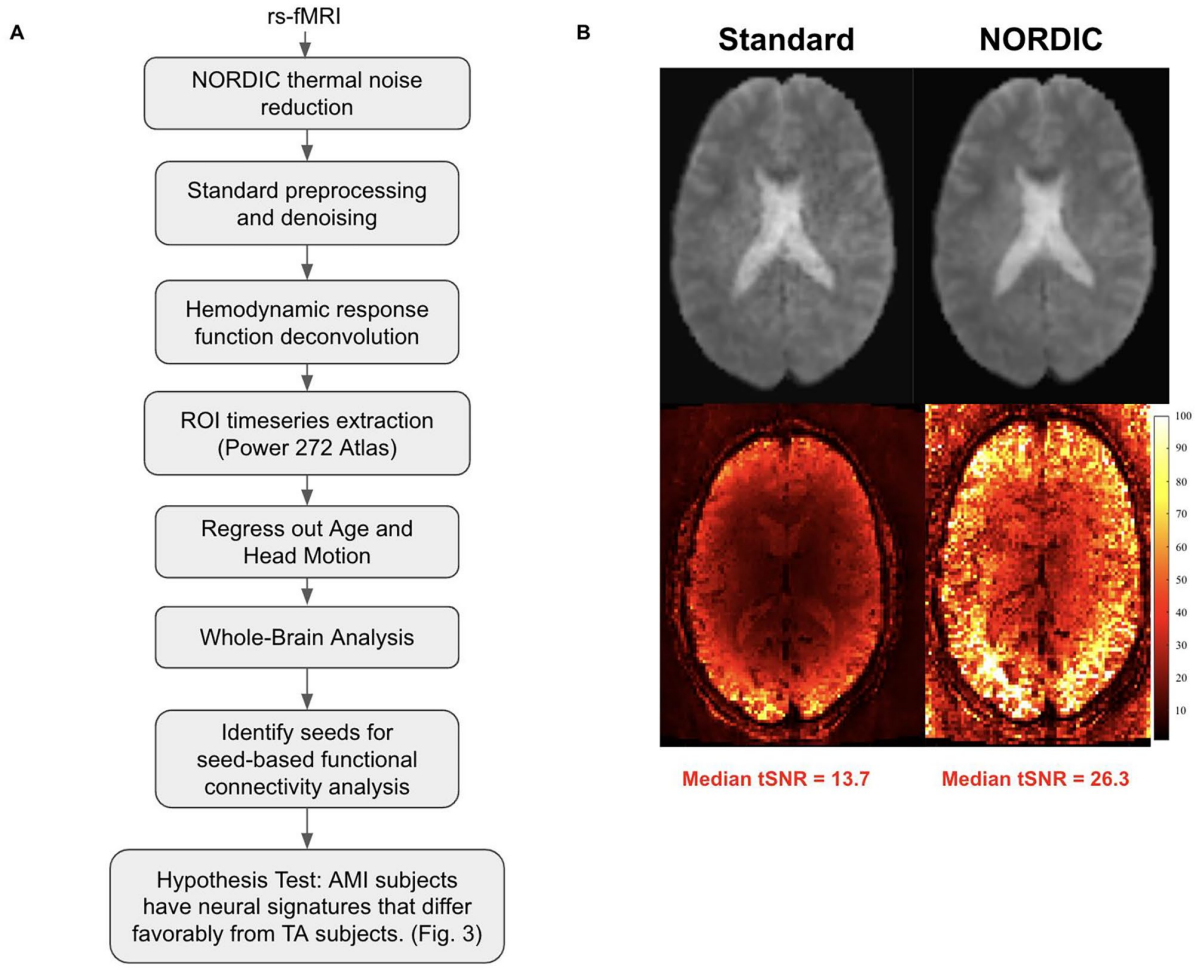
Analysis pipeline and results from NORDIC denoising. **(A)** Flowchart of the signal processing and analysis pipeline. **(B)** (Top) Example functional slice from subject 2 before and after NORDIC denoising. (Bottom) Temporal signal to noise ratio (tSNR) of the same slice from subject 2 before and after NORDIC denoising. Median tSNR after NORDIC increases by a factor of two compared to standard preprocessing before NORDIC, from 13.7 to 26.3.

An overview of the methodology applied to the raw rsfMRI data to arrive at the stage of answering our hypotheses based on group-level inferences is presented in Fig. 4A. At the onset, a signal processing approach called NOise Reduction with DIstribution Corrected (NORDIC) principal component analysis was applied to the raw rsfMRI data^39^. This denoising algorithm addresses the signal-to-noise and functional contrast-to-noise ratios by selectively suppressing the thermal noise contribution present in the fMRI data. In this case, the NORDIC technique reduced the noise by an average of 2–3 times per random voxel and doubled the median temporal signal to noise ratio (Fig. 4B), drastically increasing the statistical power of the data.

Afterwards, standard fMRI preprocessing steps were performed in the CONN toolbox version 21, which is based on SPM12^40^: functional realignment, slice-timing motion correction, ART-based outlier identification, normalization to MNI152 standard space, and spatial smoothing (Gaussian filter with a 2-mm full-width-at-half-maximum kernel). The residual BOLD signals were filtered through a 0.01 Hz high-pass filter in order to eliminate low-frequency drift often attributed to physiological noise or subject motion. Following this, linear detrending and denoising were done with 5 principle components and their derivatives of white matter and cerebrospinal fluid signals, as well as 6 motion parameters and their derivatives. Quality control was performed on the processed images and no subjects were removed. Quality control results such as those of motion correction and denoising are shown in Fig. S3. Any further denoising and analysis was performed in MATLAB^41^. Estimation and deconvolution of the hemodynamic response function (HRF) was performed^42^ to recover the rsfMRI signal without potential confounds from HRF variability on FC estimation^43^ and FC group differences^44,45^.

### Structural image parcellation

Regional time series from functionally homogeneous ROIs were extracted from the deconvolved BOLD signals using the Power 272 Atlas, which remains a competitive parcellation performer using the distance-controlled boundary coefficient^46^*.* The 272 ROIs are comprised of 242 cortical ROIs from the Power-Petersen atlas^47^, 16 subcortical ROIs from the Harvard–Oxford structural atlas^48–51^ and 14 cerebellar ROIs from the Buckner cerebellar atlas^52^.

### Exploratory whole-brain analysis

Since this was an exploratory analysis, a data-driven approach was used to identify which ROIs to use for SBC analysis. Without a priori knowledge of potentially relevant regions across the whole brain, we chose to perform a whole-brain connectivity analysis. Thereafter, upon identifying relevant regions from the whole-brain analysis, we considered what could be a next suitable step; possible options were seed-based connectivity analysis and complex network modeling based on graph theory. Being the first study of its kind, we chose to pick the simpler and more robust approach. Hence, we chose the standard seed-based connectivity analysis, and for the same reason we also chose to investigate FC based on Pearson’s correlation rather than other sophisticated approaches such as effectivity connectivity or FC based on other quantifying metrics. Future analyses of these data may use more sophisticated analytical approaches.

First, the FC, or Pearson’s zero-lag cross correlation coefficient, between each of the 272 ROIs was computed for each subject in each group. In the following statistical analyses, both head motion and age were extracted and utilized as covariates. Framewise displacement was calculated for each subject and subsequently used as the regressor representing head motion. Then, a three-way ANOVA test (p^unc^ < 0.001) was applied to identify significant differences between the AMI, TA or control groups. Finally, paired *t*-tests (*p* < 0.05, Bonferroni correction) were performed only within the significant correlations from the prior ANOVA test in order to find relevant pairwise correlations (Fig. 2A, B). Parametric testing was performed after ensuring data normality post-denoising (Fig. S3).

In both AMI vs. TA and TA vs. control comparisons, there was one particular ROI associated with the largest number of significant pairwise connections (highest node degree)^53^. Since this ROI had the highest node degree in both the comparisons, we inferred that the ROI had unique, as well as widespread, alterations in TA. This was not the case for any other ROIs. Thus, we selected it as a seed in further analysis. The ROI in question (MNI coordinates:^5,22,35^, spherical radius = 10 mm) lies on a cingulate sub-region, which is specifically associated with the salience network. Thus, for convenience we have called this ROI the “salience network seed” throughout the main text of this manuscript, though it is not to be confused with the entirety of the salience network. The BrainNet Viewer software was used for the visualization of the connectivity results from the exploratory whole-brain analyses, as well as for visualizing the selected seed ROIs in a brain volume^54^.

### Seed-based connectivity analysis

The SBC analysis was performed using the CONN toolbox*.* To assess group level FC differences, a between-subjects contrast of [− 1 1 0] was applied to the general linear model for each between-groups comparison, while simultaneously regressing out the effects of age. The SBC analysis was performed using the salience network seed. The level of FC reported corresponds to the *z*-score, or the Fisher-transformed bivariate correlation coefficient, between the average of the BOLD time series within the ROI and the BOLD time series of each independent voxel. The statistically significant cluster-level inferences obtained were labeled according to the Harvard–Oxford and AAL atlases that are default in the CONN toolbox.

### Supplementary Information

Supplementary Information.

## Data Availability

Study data may be obtained from the corresponding author upon reasonable request.
