# Supplementary material for: Resting state neurophysiology of agonist–antagonist myoneural interface in persons with transtibial amputation
Source: Sci Rep. 2024 Jun 12;14:13456. doi: 10.1038/s41598-024-63134-4 (PMC11166995; doi:10.1038/s41598-024-63134-4)
Supplement: Supplementary file 1 — Supplementary Information. [file 41598_2024_63134_MOESM1_ESM.pdf]

Supplementary Analysis

We conducted a supplementary analysis with a “motor cortex seed” that lies partly in the supplementary motor area and partly in the premotor cortex (Fig. S1).

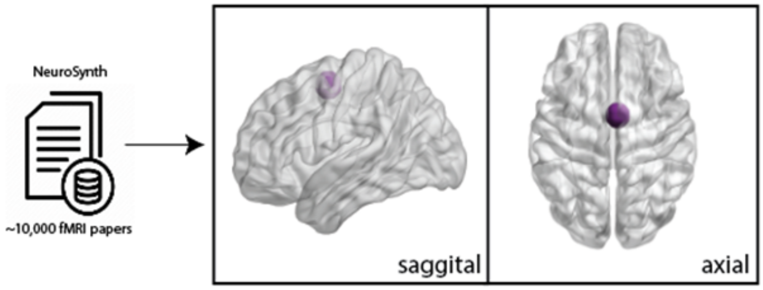

Fig S1. The motor cortex seed (spherical radius = 10 mm) as defined by a meta-analysis of neuroimaging studies in Neurosynth, located partly on the supplementary motor area and partly on the prefrontal cortex.

This motor seed was chosen due to the prominent effects of functional reorganization following lower limb amputation, and their resulting clinical impact *(1)*. The ROI was selected based on a meta-analysis identifying 438 neuroimaging studies that used the particular keywords “motor cortex” in NeuroSynth *(2)*, a large-scale database of mappings between neural and cognitive states. This motor cortex seed (MNI coordinates: (4, -2, 54); spherical radius = 10 mm) is anatomically located superior and posterior to the salience seed. We then carried out further SBC analyses with this seed. One significant cluster-level inference (size = 211 voxels) was made when examining FC with the motor cortex seed in AMI vs. TA group comparisons ($p^{unc} < 0.001$ cluster-defining threshold, $p^{FDR} < 0.001$ cluster-level threshold). This cluster (Fig. S2) was located on portions of the postcentral gyrus, superior parietal lobe and supramarginal gyrus, in a region that collectively comprises the dorsal attention stream. Thus, the

analysis reveals decreased FC between the motor cortex seed and the dorsal attention stream in AMI subjects.

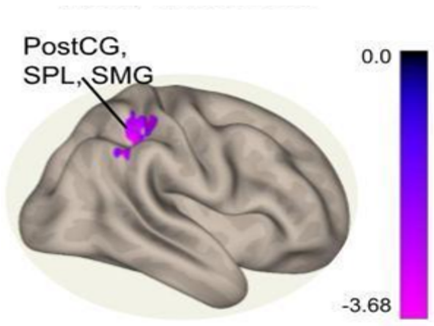

Fig S2. The dorsal attention cluster demonstrated stronger connectivity with the motor cortex seed in the TA group.

In other words, we observed decreased connectivity between the motor cortex seed and a cluster that lies on several distinct regions that collectively comprise the dorsal attention stream in AMI subjects. The dorsal attention stream is responsible for integrating information for executing immediate movements (*3*). As such, the relationship between these clusters may imply that AMI subjects consolidate less data while engaging in movements necessitating faster reaction times, which would make sense due to the restoration of afferent feedback and proprioception provided by this surgical architecture. We would like to acknowledge that there are limitations to this discussion as the motor cortex seed determined from our post hoc meta-analysis is not completely bilateral such that the seed encompasses similar altered processing in each amputee. More data is required to further investigate and validate these speculations.

Supplementary Figures

Group	Sex	Etiology	Age at Amputation (years)	Age at Scan (years)	Amputation to Scan Time (months)	Dominant Side	Amputated Side
AMI	M	Trauma	52	55	20	R	L
	M	Trauma	36	27	12	R	L
	M	Trauma	52	52	6	R	R
	F	Iatrogenic	65	67	10	R	L
	F	Iatrogenic	20	21	6	L	L
	F	Thermal Injury	41	42	13	L	L
	F	Trauma	28	28	6	R	R
	M	Malformity	36	37	14	L	L
	M	Trauma	52	53	17	R	L
	M	Vascular	41	42	13	R	L
	M	Trauma	26	28	18	R	L
	F	Trauma	50	52	26	R	L
TA	F	Trauma	59	61	20	R	R
	M	Malformity	23	25	24	R	L
	F	Trauma	58	59	10	R	L
	M	Trauma	58	60	23	R	R
	M	Trauma	56	58	17	R	L
	M	Trauma	36	39	30	R	R
	M	Trauma	57	59	22	R	R
Biologically Intact Controls	F	-	-	21	-	L	-
	M	-	-	54	-	L	-
	M	-	-	31	-	L	-
	F	-	-	73	-	R	-
	F	-	-	45	-	R	-
	M	-	-	44	-	R	-
	F	-	-	37	-	R	-
	F	-	-	32	-	R	-
	F	-	-	22	-	R	-
	F	-	-	24	-	R	-

Table S1. Subject Demographics. Information is included on sex, etiology, age, amputation to scan time, and laterality.

Salience network seed

ROI spherical radius = 10mm

Cluster Region	Centroid (MNI Space)	Cluster Size	Cluster-defining p-unc	Cluster-level p-FDR
**AMI > Control**				
LOC, TOFusC, OFusG, Cereb	(+46, -70, -06)	360	0.000017	<0.000001
SMG, PostCG, PO	(-56, -22, +30)	337	0.0002	<0.000001
LOC	(-50, -80, +02)	177	0.00022	0.00023
PP, IC, CO	(-50, +00, -04)	149	0.00038	0.00093
SMG, PO	(+60, -26, +40)	145	0.00035	0.00095
**TA > Control**				
Forb, TP, FP	(-48, +32, -14)	306	0.00001	0.000001
LOC, OP	(-20, -86, +38)	235	0.00049	0.000015
MidFG, PreCG	(-44, +10, +44)	162	0.00015	0.00057

Motor cortex seed

ROI spherical radius = 10mm

Cluster Region	Centroid (MNI Space)	Cluster Size	Cluster-defining p-unc	Cluster-level p-FDR
**AMI > Control**				
PreCG, PostCG, TP	(-56, +02, -06)	370	0.00002	<0.000001
PreCG, PostCG	(+56, -02, +46)	279	0.000013	0.000002
PreCG	(-10, -16, +68)	194	0.000007	0.000089
SMG, PostCG	(+60, -22, +38)	174	0.0003	0.00021
**TA > Control**				
FP, PaCiG, SFG	(-18, +58, +24)	340	0.000017	<0.000001
OP, LOC	(+24, -94, +20)	199	0.000011	0.00011
SFG	(-04, +36, +50)	184	0.000023	0.00017
FP	(+18, +62, +24)	174	0.000003	0.00023
LOC	(-56, -68, +08)	160	0.00037	0.00042
AC, SMA, PaCiG	(+10, +08, +46)	146	0.000003	0.00081

Table S2. Table of all remaining significant cluster-level differences (cluster-level threshold: $p^{FDR} < 0.001$, cluster-defining threshold: $p^{unc} < 0.001$) from the resting state SBC analysis. The top and bottom halves of the table contain ROIs in relation to their FC with the salience network and motor cortex seeds, respectively. Enumerated in the table is the region in which a given cluster is situated, based on the Harvard-Oxford atlas default in the CONN toolbox, each cluster's centroid in MNI space, each cluster's size, as well as the cluster-level and cluster-defining p-value thresholds. Abbreviations: FP = frontal pole, FO = frontal operculum, SMG = supramarginal gyrus, AG = angular gyrus, OP = occipital pole, LOC = lateral occipital cortex, TOFusC = temporal occipital fusiform cortex, OFusC = occipital fusiform gyrus, Cereb = cerebellum, PostCG = postcentral gyrus, PO = parietal operculum, PP = planum polare, IC = insular cortex, CO = central operculum, Forb = frontal orbital cortex, TP = temporal pole, MidFG = middle frontal gyrus, PreCG = precentral gyrus, SPL = superior parietal lobe, PaCiG = paracingulate gyrus, SFG = superior frontal gyrus, AC = anterior cingulate.

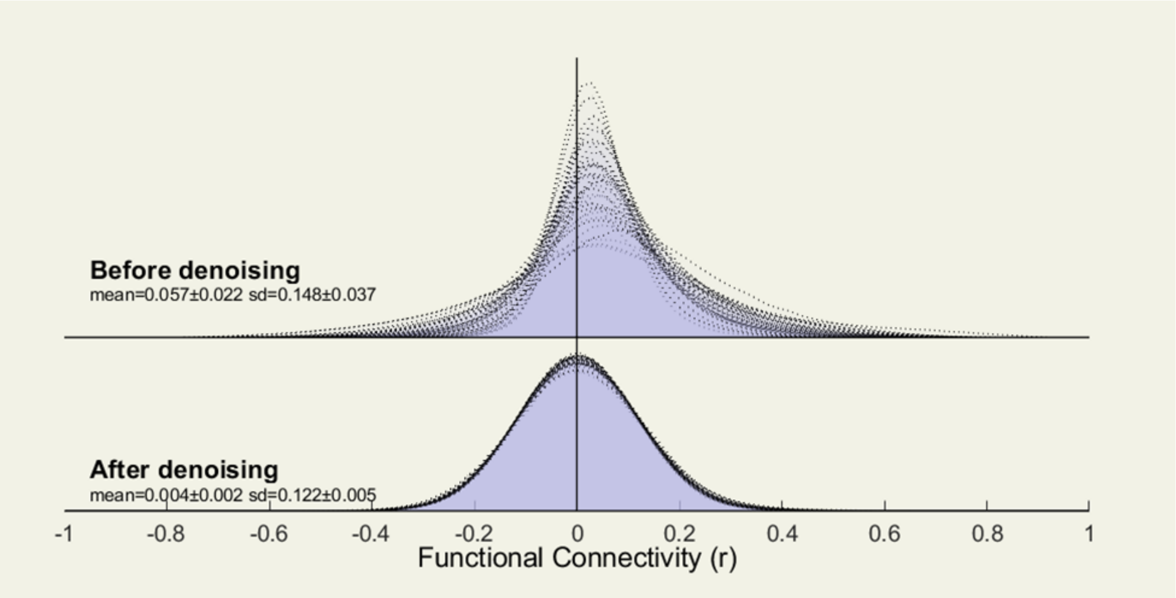

Fig S3. Quality assurance plot generated by the CONN toolbox, illustrating the distribution of FC values before and after temporal denoising.

Supplementary Bibliography

1. Rueda, F. *et al.* Knee and hip internal moments and upper-body kinematics in the frontal plane in unilateral transtibial amputees. *Gait. Posture.* **37**, 436–439 (2013).

2. Yarkoni, T., Poldrack, R., Nichols, T., Van Essen, D. & Wager, T. Large-scale automated synthesis of human functional neuroimaging data. *Nat. Methods.* **8**, 665–670 (2011).

3. Brown, J. Visual streams and shifting attention. Prog. *Brain Res.* **176**, 47–63 (2009).
